# Rifabutin-induced severe panuveitis: an unusual case of early onset in a patient with AIDS

**DOI:** 10.5935/0004-2749.202100118

**Published:** 2021

**Authors:** Ana Luiza Biancardi, Dayvison Francis Saraiva Freitas, Felipe Moreira Ridolfi, Flávia Marinho Sant´Anna, Andre Luiz Land Curi

**Affiliations:** 1 Laboratory of Infectious Diseases in Ophthalmology, Instituto Nacional de Doenças Infecciosas Evandro Chagas, Fundação Oswaldo Cruz, Rio de Janeiro, RJ, Brazil; 2 Clinical Research Laboratory on Infectious Dermatology, Instituto Nacional de Doenças Infecciosas Evandro Chagas, Fundação Oswaldo Cruz, Rio de Janeiro, RJ, Brazil; 3 Clinical Research Laboratory on Mycobacteria, Instituto Nacional de Doenças Infecciosas Evandro Chagas, Fundação Oswaldo Cruz, Rio de Janeiro, RJ, Brazil

To the Editor,

Drug-induced uveitis is a rare condition and usually based on a temporal relationship between drug use and the occurrence of uveitis. Some drugs, such as rifabutin, are well-known causative agents of uveitis. The pathogenesis is unclear, but the disease seems to be associated with direct toxicity. A non-granulomatous anterior uveitis is the most common presentation. Although the prognosis is good, atypically severe cases can occur^(1,2)^.

A 38-year-old woman with AIDS, disseminated sporotrichosis, and pulmonary tuberculosis complained of blurred vision and ocular pain in both eyes (OU) at the start of the maintenance phase of tuberculosis treatment. Since her antiretroviral therapy included a protease inhibitor (atazanavir plus ritonavir), she was unable to use rifampicin (due to drug interaction) and had to start on rifabutin. However, the patient mistakenly took rifabutin at 450 mg/day, instead of the prescribed 150 mg/day, for one week due to misunderstanding. Of note, the Brazilian standard tuberculosis treatment consists of 2 months’ (intensive phase) combination therapy of rifampicin (600 mg), isoniazid (300 mg), pyrazinamide (1500 mg), and ethambutol 1200 mg, followed by a 4-month (maintenance phase) of rifampicin (600 mg) plus isoniazid (300 mg). Laboratory tests revealed a low CD4 count (106 cells/mm^3^) and an undetectable viral load (<40 copies/mL). Serology for syphilis was negative, and complementary exams, including complete blood count, inflammatory markers, and biochemical parameters, were normal. The patient’s body weight was 52 kg, and her past medical history included a 1-month hospitalization due to disseminated sporotrichosis, which was treated with amphotericin B deoxycholate (50 mg/day for 25 days) and posaconazole (800 mg/day for 20 days) 5 months before the ocular complaint.

**Figure 1:**
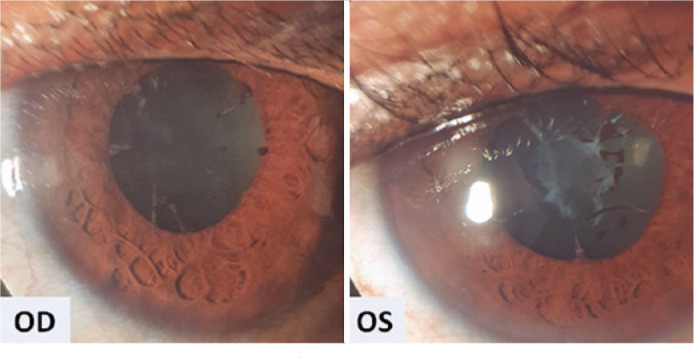
Anterior uveitis sequelae with persistence of posterior synechia and pupillary membrane. OD: Right eye; OS: Left eye.

**Figure 2:**
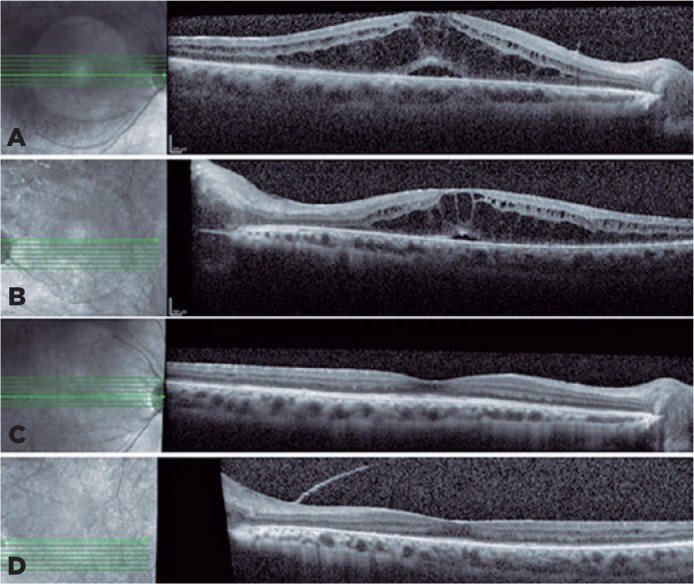
Macular edema in OD (A) and OS (B). Resolution of macular edema OD (C) and OS (D); a partially detached posterior hyaloid membrane is seen in the OS. The media opacity compromised the quality of the images. OD: Right eye; OS: Left eye.

Ophthalmic evaluation revealed visual acuity of counting fingers, biomicroscopy with 4+ anterior chamber cells, 3+ flare, posterior synechiae, and no keratic precipitates in OU. Intraocular pressure was normal, and fundoscopy was difficult to perform due to posterior synechiae and media opacity. As the abnormalities were noted, and the suspicion of severe panuveitis was considered, we promptly initiated treatment with topical dexamethasone (1 mg/mL every 2 hours) and tropicamide (1 mg/mL every 8 hours) with slow withdrawal for 30 days. In addition, the rifabutin dose was adjusted (150 mg/day). After 30 days, anterior chamber inflammation improved (Figure 1). Fundoscopy showed vitreous opacities OU and inferior vasculitis in the left eye. However, the patient’s visual acuity remained low, and an optical coherence tomography revealed macular edema OU (Figures 2A and 2B). After tapering the topical corticosteroid and adjusting the rifabutin, full recovery of panuveitis was achieved after two months (Figures 2C and 2D).

The present case describes an unusual presentation of rifabutin-induced panuveitis with early occurrence. In previous reports, especially on anterior uveitis, the onset is between 2 weeks and 12 months after rifabutin initiation^(1)^. Concomitant use of ritonavir is associated with increased risk due the inhibitory effect on CYP3A, which is essential in the metabolism of rifabutin and increases its serum levels^(1)^. The incidence of uveitis is also associated with low body weight. Shafran et al.^(3)^ reported an incidence of 64% of anterior uveitis in patients weighing <55 kg. In the present case, the use of ritonavir and the patient’s body weight may have contributed to panuveitis. Moreover, daily doses of rifabutin ranging from 300 to 1,800 mg is also associated with uveitis, and its occurrence and severity are influenced by the dosage and duration of treatment^(1-3)^. A previous study evaluated 59 HIV-positive patients prescribed with rifabutin at 600 mg/day and reported anterior uveitis in 39% of the patients, in average 65 days (range, 27-197 days) after starting treatment^(4)^. However, in the present case, panuveitis occurred earlier.

The major concern was to exclude opportunistic diseases as the causative factor, but some considerations must be raised. First, the temporal relationship between the increased dosage of rifabutin and the occurrence of panuveitis is well established. Second, with rifabutin dose adjustment and the use of topical steroids and mydriatic drugs, ocular inflammation resolved without any other changes in the treatment of tuberculosis or sporotrichosis. Additionally, severe panuveitis due to other causes would not resolve without specific treatment and/or oral corticosteroids. Lastly, no association between panuveitis with immune recovery syndrome occurred, as the CD4 count did not change.

To the best of our knowledge, only one report on rifabutin-induced panuveitis has been published, corroborating its rarity^(5)^. Furthermore, although rare, severe rifabutin-induced uveitis can occur prematurely, which physicians should bear in mind in clinical practice.
